# The Prospective Predictive Power of Parent-Reported Personality Traits and Facets in First-Onset Depression in Adolescent Girls

**DOI:** 10.1007/s10802-024-01186-w

**Published:** 2024-03-19

**Authors:** Yiming Zhong, Greg Perlman, Daniel N. Klein, Jingwen Jin, Roman Kotov

**Affiliations:** 1 Department of Psychology, The University of Hong Kong, Hong Kong SAR, China; 2 State Key Laboratory of Brain and Cognitive Sciences, The University of Hong Kong, Hong Kong SAR, China; 3 Department of Psychiatry, Renaissance School of Medicine, Stony Brook University, Stony Brook, NY USA; 4 Department of Psychology, Stony Brook University, Stony Brook, NY USA

**Keywords:** Parent-report, Self-report, Personality, Adolescent, First-onset depression

## Abstract

Certain personality traits and facets are well-known risk factors that predict first-onset depression during adolescence. However, prior research predominantly relied on self-reported data, which has limitations as a source of personality information. Reports from close informants have the potential to increase the predictive power of personality on first-onsets of depression in adolescents. With easy access to adolescents’ behaviors across settings and time, parents may provide important additional information about their children’s personality. The same personality trait(s) and facet(s) rated by selves (mean age 14.4 years old) and biological parents at baseline were used to prospectively predict depression onsets among 442 adolescent girls during a 72-month follow-up. First, bivariate logistic regression was used to examine whether parent-reported personality measures predicted adolescent girls’ depression onsets; then multivariate logistic regression was used to test whether parent reports provided additional predictive power above and beyond self-reports of same trait or facet. Parent-reported personality traits and facets predicted adolescents’ depression onsets, similar to findings using self-reported data. After controlling for the corresponding self-report measures, parent-reported higher openness (at the trait level) and higher depressivity (at the facet-level) incrementally predicted first-onset of depression in the sample. Findings demonstrated additional variance contributed by parent-reported personality measures and validated a multi-informant approach in using personality to prospectively predict onsets of depression in adolescent girls.

**Supplementary Information:**

The online version contains supplementary material available at 10.1007/s10802-024-01186-w.

Adolescence is a critical developmental period for first-onset depressive disorders (Kessler & Bromet, 2013; Rice et al., 2019), as approximately 15%-20% of individuals with depression in adulthood have onsets between the age of 12 and 19 (Kessler et al., 2005; Rohde et al., 2009). Compared to those with an adult onset, adolescence-onset depression is associated with more severe outcomes, including educational failure, unemployment, problematic marital and social relationships, and suicide (Bodden et al., 2018; Clayborne et al., 2019). Females are twice as likely to develop depression than males, and such sex differences typically emerge and enlarge during adolescence. Moreover, compared to boys, depression in girls may be more persistent, with longer durations and more episodes. (Breslau et al., 2017; Essau et al., 2010). It is therefore imperative to investigate risk factors that can prospectively predict first onset depression, particularly in girls, which will facilitate identifying at-risk youngsters for prevention and early intervention.

The Big Five framework is a widely accepted taxonomy for individual differences in personality (Costa & McCrae, 1995; Goldberg, 1993), and is often used in research on the relationship between personality and psychopathology, including depression (Klein et al., 2011; Kotov et al., 2010; South et al., 2010; Watson & Naragon-Gainey, 2014). Personality continuity is largely maintained during adolescence (Roberts et al., 2001, 2006). Thus far, only a handful of studies have examined the associations of Big Five traits with first onset of depression prospectively (Goldstein et al., 2018; Kendall et al., 2015; Michelini et al., 2021; Zinbarg et al., 2016). These studies have shown that self-reported higher neuroticism, lower extraversion, and lower conscientiousness predicted first-onset depression during adolescence. At the lower-order facet level, self-reported higher depressivity and anxiousness (neuroticism facets), lower positive emotionality and sociability (extraversion facets), and lower self-discipline (conscientiousness facet) prospectively predicted first-onset of depression (Goldstein et al., 2018; Michelini et al., 2021; Zinbarg et al., 2016).

In most studies on the relationship between personality and depression, self-report has been used as the sole source of personality information. Self-reports have many advantages including accessibility, efficiency, as well as information richness (McDonald, 2008; Paulhus & Vazire, 2007), and it has informed clinical assessment, treatment planning, and prediction of outcome (Stanton et al., 2019). However, self-reports are prone to bias, influenced by social and situational contexts, and can be confounded by the participant’s response style and mood state (Chmielewski & Watson, 2009; Klein et al., 2011). For example, individuals who report high levels of neuroticism and extraversion tend to over-report their experiences of negative and positive emotions, respectively (Fossum & Barrett, 2000). In addition, social desirability and acquiescent responding styles might further contribute to biased self-report (Paulhus & Vazire, 2007; Soto et al., 2008). Moreover, if the participant is the source of information regarding both personality and depression, it can inflate associations due to shared method variance (Klein et al., 2011).

Multi-informant assessment approaches have long been used in developmental psychopathology research to address the limitations of self-report, and to collect information on behavior that may be context-sensitive (e.g., home versus school) (Lapouse & Monk, 1959; Achenbach et al., 1987, 2005; Hunsley & Mash, 2007; McDonald, 2008; De Los Reyes et al., 2022). In a multi-informant assessment approach, information is typically gathered from informants such as parents, teachers, and peers, who can observe the youth in a range of naturalistic environments, thereby providing richer information than self-report only (Achenbach et al., 1987; De Los Reyes et al., 2022; Kraemer et al., 2003). Prior studies revealed that informant-report of personality provides unique variance and thereby incremental predictive validity in life outcomes, including academic achievement and job performance (Connelly & Ones, 2010). The incremental validity of parent-report in predicting clinical outcomes during assessments of youth’s psychosocial functioning has also been widely demonstrated (De Los Reyes & Ohannessian, 2016; Dirks et al., 2012). For example, parents’ ratings of their child’s openness and conscientiousness provided incremental variance beyond self-report in predicting academic adjustment, while parents’ ratings of neuroticism independently predicted emotional adjustment outcomes (Kurtz et al., 2012).

While both self-report and informant-report contribute to the prediction of psychopathology, their relative predictive power varies by the specific trait and facet (Jones & Miller, 2012; Markon et al., 2013; Lieberman et al., 2016). For example, self-reported neuroticism and extraversion were found to predict subjective emotional experiences better than corresponding informant-report (Spain et al., 2000). In contrast, self-report was less informative than informant-report in predicting the associations between impulsive and antagonistic traits and psychopathology (Gauthier et al., 2009; Quilty et al., 2018). In the case of predicting first-onset depression during adolescence, parents usually observe their children across different situations. Thus, including parent report of personality has the potential to provide incremental predictive power with respect to depression onset.

It remains unknown whether parents’ reports of adolescents’ personality traits and facets predict subsequent depression onsets prospectively. Building on existing knowledge of known personality risk factors for adolescent depression using self-report (Goldstein et al., 2018), the present study examined 1) the prospective prediction of parent-report; 2) its relative validity compared to self-report; and 3) its incremental validity above and beyond self-report, in a large sample of adolescent girls. Based on prior evidence using self-reported personality measures in Goldstein et al. (2018), we hypothesized that the same personality traits (neuroticism, conscientiousness, extraversion) and facets (depressivity, anxiousness, positive emotionality, sociability, self-discipline, deliberateness), as reported by parents, would prospectively predict the first-onset of depression. In addition, we hypothesized that parent-report of traits and facets would account for unique variance over and above self-report in predicting first-onset of depression. For the neuroticism domain, while it is less observable, parent-report may provide additional information less affected by biased reporting style and mood congruency pertinent to self-report (Fossum & Barrett, 2000; Klein et al., 2011; Paulhus & Vazire, 2007). For the extraversion domain, parent-report may supplement self-report given high observability of this domain (Vazire, 2010). Given the relatively high social desirability embedded in conscientiousness, agreeableness, and openness domains (Vazire, 2010), parents may provide supplementary information to make up for possible self-reporting biases pertinent to these trait domains (Achenbach et al., 1987). We also examined the facets of neuroticism, extraversion and conscientiousness given prior evidence of their predictive validity for adolescent’s first onset depression (Goldstein et al., 2018), and extended the same hypotheses regarding the incremental predictive power of parent-report for these facets.

## Materials and Methods

### Participants

Five hundred and fifty adolescent girls aged between 13.5 and 15.5 years old (*M* = 14.4, *SD* = 0.6) and one of their biological parents (93.1% mothers) aged between 31 and 59 years old (*M* = 46.5, *SD* = 4.5) participated in the study, as measured at wave one (baseline) assessment. Amongst the 550 adolescents, 80.7% were non-Hispanic White, and 33.6% had both parents graduated from college (Goldstein et al., 2018; Michelini et al., 2021). Of these, 67 missed the wave six assessment (detailed below), six developed bipolar disorders and one developed depression due to general medical condition, hence they were excluded from data analyses; 34 adolescents who were diagnosed with Depression Not Otherwise Specified (NOS) at baseline were also excluded from the data analysis. Missing items on questionnaires were addressed using ipsative mean imputation (Schafer & Graham, 2002) if at least 80% of items on the scale were completed. Participants with missing outcomes were excluded from analyses, because we could not accurately infer depression onset from data available.

After removing the 108 girls, 442 adolescent girls remained in the study. At the wave one assessment, the 442 adolescent girls were between 13 and 16 years old (*M* = 14.4, *SD* = 0.6). At the wave six assessment, they were between 19 and 23 years old (*M* = 20.3, *SD* = 0.9). At wave one, 89.6% of them were non-Hispanic White, and 34.6% had both parents graduated from college. We compared demographic factors and baseline personality measures between the included 442 adolescents (completers) and the 108 excluded adolescents (attritors). There were no significant differences in demographic factors except for parents’ education level, where completers had higher number of both parents graduated from college χ^2^(2) = 8.25, *p* = 0.016).

Comparing baseline personality measures between completers and attritors (see Supplementary Table 1), small differences were found in personality measures reported by youth and parents, respectively (Cohen's *d*’s ranging from -0.38 to 0.33). For neuroticism and depressivity, completers’ ratings were significantly lower than attritors’ ratings for both self- and parent-reports (*ps* range from < 0.001 to 0.012). For dutifulness and orderliness, completers’ ratings were significantly higher than attritors’ ratings for both self- and parent-reports (*ps* range from 0.003 to 0.030). For conscientiousness, achievement and self-discipline, completers’ ratings were significantly higher than attritors’ ratings for parent-report only (*ps* range from 0.002 to 0.012).

Participants were recruited from the community using a variety of channels, including commercial mailing lists, advertisement, referrals, and word-of-mouth, as part of Adolescent Development of Emotions and Personality Traits (ADEPT) project conducted by Stony Brook University, New York. As the goal of the ADEPT project was to examine risk factors predicting first-onset depression during adolescence, adolescents with a history of major depressive disorder (MDD) or Dysthymia were excluded. This was achieved through the lifetime version of Patient Health Questionnaire (PHQ-9; Cannon et al., 2007) during telephone screening and followed by the Kiddie Schedule for Affective Disorders and Schizophrenia for School-Age Children, Present and Lifetime Version (K-SADS-PL; Kaufman et al., 1997), a semi-structured diagnostic interview administered with the adolescent. Other eligibility criteria included fluency in English, ability to complete questionnaire, and availability of at least one biological parent. Written assent and consent were obtained from all adolescents and their biological parents in-person at the beginning of the study.

### Procedure

At the baseline in-person visit (wave one), each adolescent and one biological parent completed personality measures assessing the youth’s traits. History of adolescents’ psychopathology was assessed using K-SADS-PL administered to the youth (Kaufman et al., 1997). Four subsequent assessments were conducted at nine-month intervals (waves two to five), and the sixth assessment was conducted 36 months later. As part of each follow-up assessment, adolescents completed the K-SADS-PL, which was conducted in-person at waves three, five, six) and by telephone at waves two and four. The current study included data from wave one to wave six (i.e., 72 months since the baseline assessment).

The ADEPT project was approved by the Institutional Review Board of Stony Brook University, and ethics approval for the current study was also obtained from the Human Research Ethnic Committee of the University of Hong Kong.

### Baseline Personality Measures

In examining the prospective association between personality measures under Big Five framework and first onsets of depression, five higher order traits were assessed using Big Five Inventory while facets under three traits domains (neuroticism, conscientiousness, and extraversion) were assessed using Faceted Inventory of the Five Factor Model. The facets of agreeableness and openness were not assessed, given majority of prior studies did not find robust associations between these facets and depression (Kotov et al., 2010).

#### Big Five Inventory

The Big Five Inventory (BFI; John & Srivastava, 1999) is a widely used 44-item questionnaire that evaluates five higher order trait dimensions: neuroticism, conscientiousness, extraversion, agreeableness, and openness. Items contained prototypical trait adjectives developed by expert ratings and factorial analytics. ADEPT dropped three items (two from extraversion and one from openness) due to corrected item-total correlations less than 0.15. The internal consistency, measured by Cronbach’s alpha, for self-reported BFI measures ranged from 0.76 to 0.83 (*M* = 0.80) (Goldstein et al., 2018), while parent-reported BFI measures ranged from 0.78 to 0.86 (*M* = 0.83).

#### Faceted Inventory of the Five Factor Model

The Faceted Inventory of The Five Factor Model (FI-FFM; Naragon-Gainey et al., 2009; Watson et al., 2019) is a questionnaire that evaluates the facets of the Big Five traits. Based on prior empirical evidence on personality trait and facet structures associated with depression (Kotov et al., 2010; Watson & Naragon-Gainey, 2014), ADEPT selected subsets of FI-FFM facets of three trait domains (neuroticism, conscientiousness, and extraversion). These facets included anxiousness (worry and apprehension propensity) and depressivity (sad, loneliness and guilt propensity) from neuroticism; achievement (setting and working towards high goals), deliberateness (careful considerations of actions), dutifulness (being reliable), orderliness (being organized and tidy), and self-discipline (ability to focus in distraction) from conscientiousness; and ascendence (pleasure in leadership/center-of-attention position), positive emotionality (energy, joyfulness and playfulness propensity), sociability (pleasure in being around people), and venturesomeness (pleasure in seeking experience stimulation) from extraversion. (Goldstein et al., 2018).

Each item was scored on a five-point scale from disagree strongly (1) to agree strongly (5). ADEPT dropped one item from the deliberation facet as it showed a corrected item-total correlation of less than 0.15. The internal consistency, measured by Cronbach’s alpha, for self-reported FI-FFM measures ranged from 0.79 to 0.87 (*M* = 0.83) (Goldstein et al., 2018), while parent-reported FI-FFM measures ranged from 0.83 to 0.91 (*M* = 0.87).

### Adolescent’s Psychopathology Assessment

The K-SADS-PL is a semi-structured diagnostic interview widely used to assess psychopathology in youth. Interviews were conducted by trained research assistants under the supervision of three clinical psychologists (RK, GP, DK). A subset of interviews was re-evaluated by a second interviewer who had no prior knowledge of the original diagnosis. A second rater independently rated audio recordings of interviews on 48 participants using the kappa statistic to establish interrater reliability, which was 0.73 for MDD and 0.85 for Dysthymia. By the 72-month follow-up, 173 of 442 adolescents had developed depressive disorders (119 MDD, 9 Dysthymia and 45 Depression NOS), compared to 269 adolescents who did not develop depression. Therefore, the 173 adolescents were classified as depressed while the other 269 adolescents were classified as non-depressed for subsequent analyses.

### Data Analysis

First, cross-informant associations between self- and parent- reported personality measures at baseline were examined using Pearson’s correlations.

Our main data analysis primarily consisted of two steps: running multiple binary logistic regression analyses and then multiple hierarchical logistic regression analyses. In the first step, we examined the predictive power of each of the baseline personality measures (self-reported and parent-reported general traits and facets) one at a time in predicting adolescents’ depression onset using binary logistic regressions. The resulting odds ratios (OR) were interpreted as the increase in odds of adolescents developing depressive disorders for each one-standard deviation increase in the personality measures. From this step of analysis, we identified the significant personality predictors for adolescents’ depression onsets. We set the alpha level at *p* < 0.01 for both self- and parent-reported traits and facets given the number of analyses, consistent with previous practice (Goldstein et al., 2018).

Next, we examined the unique contribution of parent reports over and above adolescents’ self-reports using hierarchical logistic regression models. We entered the parent-reported personality measures that had shown bivariate associations with depression at *p* < 0.01 in block 2, after self-report of the same trait or facet was entered in block 1. Regression coefficients were used to determine the relative predictive power, and the change in chi-square from block 1 to block 2 was used to determine the incremental predictive power of adding the parent-report measures.

## Results

### Associations Between Parent- and Self-Reported Personality Measures

Table 1 shows Pearson’s correlation coefficients between self- and parent-reported personality ratings of the adolescents at baseline, all of which were statistically significant (*p* < 0.001). At the higher-order trait level, the correlations ranged from 0.38 (agreeableness) to 0.63 (extraversion). At the facet level, the correlations ranged from 0.30 (deliberateness) to 0.59 (orderliness and ascendence).

**Table 1:** Baseline Cross-informant Traits, Facets Correlations

Personality Measures	Self-report		Parent-report		Cross-informant
	M	SD	M	SD	*r*
Neuroticism	2.7	0.8	2.7	0.9	0.49***
Anxiousness	2.9	0.9	2.7	0.9	0.46***
Depressivity	2.1	0.8	1.8	0.8	0.37***
Conscientiousness	3.7	0.7	3.8	0.8	0.54***
Achievement	4.5	0.6	4.2	0.7	0.48***
Deliberateness	3.6	0.7	3.6	0.9	0.30***
Dutifulness	4.3	0.6	4.3	0.7	0.41***
Orderliness	3.6	0.9	3.5	1.0	0.59***
Self-discipline	3.4	0.8	3.7	0.9	0.49***
Extraversion	3.8	0.8	3.6	0.9	0.63***
Ascendence	3.4	0.9	3.4	1.0	0.59***
Positive Emotionality	4.1	0.7	4.0	0.7	0.47***
Sociability	3.8	0.7	3.8	0.8	0.50***
Venturesomeness	4.1	0.7	3.9	0.8	0.44***
Agreeableness	4.1	0.6	4.2	0.7	0.38***
Openness	3.9	0.6	4.0	0.6	0.41***

*p < 0.05; **p < 0.01; ***p < 0.001

### Traits Predicting Adolescents’ Depression Onset

Bivariate correlations are shown in Table 2. For self-report, consistent with previous findings for the first 18 months of the study (Goldstein et al., 2018), adolescents’ depression first-onsets were predicted by higher neuroticism and lower conscientiousness, extraversion, and agreeableness at the trait level. At the facet level, also aligned with Goldstein et al. (2018), adolescents’ depression first-onsets were predicted by higher anxiousness and depressivity (from neuroticism), lower positive emotionality and sociability (from extraversion), and lower orderliness, deliberateness, dutifulness, and self-discipline (from conscientiousness).

**Table 2:** Bivariate Self- and Parent-reported Trait and Facet Predictors of Adolescents’ Depression First-onsets from Baseline to 72-month Follow-up

Personality Measures	Self-report			Parent-report		
	OR	95% CI		OR	95% CI	
Neuroticism	2.03***	(1.55	2.65)	1.51***	(1.21	1.88)
Anxiousness	1.66***	(1.32	2.10)	1.43***	(1.15	1.78)
Depressivity	1.93***	(1.50	2.49)	1.66***	(1.31	2.12)
Conscientiousness	0.47***	(0.34	0.64)	0.79*	(0.62	1.00)
Achievement	0.74	(0.54	1.01)	0.87	(0.68	1.13)
Deliberateness	0.62***	(0.47	0.81)	0.78*	(0.62	0.98)
Dutifulness	0.49***	(0.35	0.70)	0.75*	(0.57	1.00)
Orderliness	0.70**	(0.56	0.87)	0.78**	(0.65	0.94)
Self-discipline	0.59***	(0.46	0.75)	0.75**	(0.61	0.92)
Extraversion	0.70**	(0.55	0.90)	0.95	(0.77	1.19)
Ascendence	0.97	(0.79	1.19)	1.05	(0.86	1.29)
Positive Emotionality	0.64***	(0.47	0.85)	0.84	(0.63	1.11)
Sociability	0.56***	(0.42	0.73)	0.84	(0.66	1.06)
Venturesomeness	0.82	(0.61	1.10)	0.96	(0.75	1.23)
Agreeableness	0.43***	(0.31	0.61)	0.85	(0.64	1.13)
Openness	1.35	(0.97	1.87)	1.69**	(1.22	2.34)

(OR = odds ratio, CI = 95% confidence intervals)

*p < 0.05; **p < 0.01; ***p < 0.001

Regarding parent-reports, adolescents’ depression first-onsets were significantly predicted by higher neuroticism and openness (*p* < 0.01), and lower conscientiousness at a trend level (0.01 < *p* < 0.05), but not extraversion or agreeableness. At the facet level, parents’ reports of both neuroticism facets (anxiousness and depressivity), and two conscientiousness facets (self-discipline and orderliness) significantly predicted adolescents’ depression first-onsets. No extraversion facet emerged as a significant predictor using parent-report.

### Parent-Reported Personality Measures in Predicting Adolescents’ Depression Onset

At the higher-order trait level (Table 3), two multivariate logistic regressions were conducted to examine the prospective independent predictive power of parent-reported traits (neuroticism and openness)—the traits that met statistical significance in association with depression first-onsets in bivariate analyses (alpha level = 0.01) – adjusting for the corresponding self-reported traits. Parent-reported higher openness remained a significant predictor of adolescents’ first-onset depression above and beyond self-reported openness, where self-report was not a significant predictor. Furthermore, parent-reported openness added incremental prospective predictive power to the corresponding self-report ΔR^2^ = 1.76%, Δχ^2^(1) = 7.85, *p* = 0.005. For neuroticism, parent-report was no longer a significant predictor after controlling for self-report.

**Table 3:** Multivariate Self- and Parent-reported Traits Predictors of Adolescents’ Depression First-onset from Baseline to 72-month Follow-up

	Openness		Neuroticism	
	OR	(95% CI)	OR	(95% CI)
*Block 1*				
Self-report	1.35	(0.97–1.87)	2.03	(1.55–2.65) ***
*Block 2*				
Self-report	1.10	(0.76–1.57)	1.87	(1.39–2.53) ***
Parent-report	1.64	(1.15–2.33) **	1.17	(0.90–1.50)

(OR = odds ratio, CI = 95% confidence intervals)

*p < 0.05; **p < 0.01; ***p < 0.001

At facet level (Table 4), four multivariate logistic regressions were conducted to examine the prospective independent predictive power of parent-reported facets (anxiousness, depressivity, orderliness, self-discipline) that survived the cut-off in the bivariate analyses (alpha level = 0.01). For depressivity, both self-report and parent-report independently predicted adolescents’ first-onset depression. Furthermore, parent-reported depressivity added incremental predictive value, ΔR^2^ = 1.19%, Δχ^2^(1) = 5.61, *p* = 0.018, above and beyond self-report. Self-reported higher anxiousness, lower self-discipline, and lower orderliness were independent risk factors in their respective analyses; however, parent-reports of the corresponding facets did not explain significant independent variance.

**Table 4:** Multivariate Self- and Parent-reported Facet Predictors of Adolescents’ Depression First-onset from Baseline to 72-month Follow-up

	Depressivity		Anxiousness		Orderliness		Self-discipline	
	OR	(95% CI)	OR	(95% CI)	OR	(95% CI)	OR	(95% CI)
*Block 1*								
Self-report	1.93	(1.50–2.49) ***	1.66	(1.32–2.10) ***	0.70	(0.56–0.87) **	0.59	(0.46–0.76) ***
*Block 2*								
Self-report	1.74	(1.33–2.27) ***	1.53	(1.18–1.98) **	0.75	(0.57–0.97) *	0.62	(0.47–0.82) ***
Parent-report	1.37	(1.06–1.78) *	1.19	(0.93–1.53)	0.91	(0.72–1.14)	0.91	(0.71–1.15)

(OR = odds ratio, CI = 95% confidence intervals)

*p < 0.05; **p < 0.01; ***p < 0.001

Together, these results showed that parent-reports of personality traits are significant predictors of adolescents’ depression first-onset, similar to adolescents’ self-reported traits. In most cases, parent reported traits did not contribute significant unique variance over and above adolescent self-reports. However, parent-reported openness trait and the depressivity facet of neuroticism exhibited independent predictive power above and beyond the corresponding self-reports in predicting first-onset depression in adolescent girls.

## Discussion

Certain personality traits and facets are risk factors for developing first-onset depressive disorders during adolescence (Goldstein et al., 2018; Kendall et al., 2015; Michelini et al., 2021; Zinbarg et al., 2016). Previous literature has predominantly relied on self-report as the sole source of personality information, while little is known about the predictive value of informant-reports of adolescents’ personality in predicting adolescent onset depression. A multi-informant assessment approach helps to mitigate the limitations inherent in the use of self-report alone (Achenbach et al., 1987; Connelly & Ones, 2010; De Los Reyes et al., 2022; Kraemer et al., 2003; Tackett, 2011; McCrae & Costa, 2004). Also, when adolescents are the information provider for both personality measures and depression symptoms, this shared source of measurements may inflate the association between personality and depression (Klein et al., 2011). Including other informants’ reports may provide incremental variance and increase the predictive power of personality with regards to depression onset.

The current study extended our previous work examining self-report personality traits and facets as predictors of adolescent girls’ first-onset depression (Goldstein et al., 2018) in several meaningful ways. First, it reinforced previous findings of self-reported personality risk factors using a much longer timeframe, which quadrupled from 18 to 72 months. As an additional finding, self-reported lower orderliness (from trait conscientiousness) emerged as another facet-level predictor. Second, it validated the prospective predictive power of parent-reported personality measures in predicting first-onset depression in adolescent girls. Similar to self-report, parent-reported higher neuroticism at the higher-order trait level, both facets of neuroticism (anxiousness and depressivity) and two facets of conscientiousness (orderliness and self-discipline) predicted adolescents’ first-onset of depression. Moreover, we found parent-reported openness at the trait level and the depressivity facet of neuroticism displayed significant independent and incremental predictive power above and beyond self-report of the corresponding trait/facet. Whereas the self-reported openness trait did not show significant bivariate association with depression onset, parent-reported higher openness significantly predicted adolescents’ depression onsets. These findings highlight the importance of a multi-informant approach by incorporating parent-report in assessing personality as a risk factor for depression.

Consistent with previous studies (Brandes et al., 2021; Göllner et al., 2017; Luan et al., 2017; Van den Akker et al., 2014), adolescents’ and parents’ reports of personality were moderately positively correlated across traits and facets. Also, in line with past literature on informant report discrepancies (Lapouse & Monk, 1959; Achenbach et al., 1987; Vazire, 2010), self-parent discrepancies were higher amongst personality traits and facets that were less observable (e.g. neuroticism, depressivity, deliberateness), and more socially desirable (e.g. agreeableness and openness). Also consistent with previous findings on personality predictors of first-onset depressive disorders (Goldstein et al., 2018; Zinbarg et al., 2016), parent-reported higher neuroticism and lower conscientiousness were significant and trending trait predictors, respectively. At the facet level, first-onset depression was significantly predicted by parent-reported higher anxiousness and depressivity from the neuroticism domain, and lower orderliness and self-discipline (as well as lower deliberateness and dutifulness at a trend level) from the conscientiousness domain. In contrast, the hypotheses regarding parent-reported lower extraversion trait and facets (e.g., positive emotionality, sociability) and lower agreeableness being significant predictors were not supported. The lack of findings involving parent-reported extraversion and agreeableness in predicting adolescents’ depression onsets might be related to the fact that adolescents spend increasingly more time away from home, reducing opportunities for parents to observe trait-relevant cues in these domains from youth’s social behaviours and interpersonal interactions (Deros et al., 2018; Rausch et al., 2017).

Notably, we found that higher parent-reported openness was a significant predictor of first-onset depression during adolescence. This was not expected, as openness was not a prospective risk factor identified in previous longitudinal studies using self-report, and previous cross-sectional studies found a negative rather than positive association (Gong et al., 2020; Khoo & Simms, 2018; Koorevaar et al., 2017). It has been posited that a lower level of openness is associated with depression because it reflects cognitive rigidity (Khoo & Simms, 2018). DeYoung et al. (2012) conceptualized openness as a paradoxical simplex which integrates aspects of intelligence and apophenia (perception of non-existent patterns or causality) that are negatively correlated with each other and posited that a very high level of apophenia could be a feature of psychopathology. However, this does not explain why parent- but not self-report had predictive value. One possible explanation might be the high social desirability in the openness trait which resulted in possible self-reporting bias, making parents the more ‘objective’ informant of this personality dimension in predicting behavioral outcomes (Vazire, 2010). This would be consistent with previous findings where informant-report on openness trait provided incremental predictive value in life outcomes compared to self-report (Bratko et al., 2006; Kurtz et al., 2012). Last but not least, openness includes aesthetic/artistic inclinations or being open to socially non-confirming attitudes and roles. The former is associated with the elevated rate of mood disorders among creative professionals, while the latter is associated with feelings of social alienation and elevated rates of depression (Gong et al., 2020; Khoo & Simms, 2018; Koorevaar et al., 2017).

Parents may have access to information about adolescents’ personalities that the adolescents are unaware of or unwilling to report, and which may predict depression onsets. We examined whether parent-reported personality traits and facets independently predicted first-onset depression above and beyond corresponding self-reports. At the higher-order trait level, parent-reported higher openness continued to account for unique variance in predicting first onsets of depression after self-report were controlled for. However, parent-reported higher neuroticism did not display significant independent predictive power. At the facet level, both self- and parent-reports of higher depressivity were significant predictors when they were entered together in the multivariate model, highlighting their unique predictive power. In contrast, only self- but not the parent-, reported anxiousness was a significant predictor of depression first-onset. Self-parent agreement on ratings of neuroticism was overall moderate during adolescence (Laidra et al., 2006), similar to self-other agreements in adulthood (Watson et al., 2000). During adolescence, parents increasingly viewed their child’s personality development more positively (Luan et al., 2017). Self-report might reflect internalisation of psychopathology by adolescents, which were not directly observable by parents (Deros et al., 2018; Vazire, 2010). On the other hand, parents might still be able to provide additional information that is less affected by youth’s mood state or reporting style at the depressivity facet level within the neuroticism domain. (Fossum & Barrett, 2000; Klein et al., 2011; Paulhus & Vazire, 2007).

Importantly, the “criterion” measure of depression onset in this study was based on youth’s self-report, which is the typical source of information for adolescents’ depression diagnosis (Goldstein et al., 2018; Michelini et al., 2021; Zinbarg et al., 2016). When the adolescent was the informant for both personality and depression criterion, it could bias the design of the study in favor of youths’ reports of personality being stronger predictors than parents’ reports because common method variance could inflate the associations between personality measures and the depression outcome (Klein et al., 2011). Given this bias, the multi-informant approach in the present study provided a particularly stringent test for the incremental validity of parent-reports of adolescent’s personality. In other words, the fact that parent-reported openness and parent-reported depressivity still showed incremental value above and beyond corresponding self-reports is noteworthy.

This study demonstrated the value of a multi-informant approach, particularly the inclusion of parent-report in addition to self-report, in examining personality risk factors for adolescent first-onset depression**.** Whilst self-reports of many of the Big Five traits and facets were effective predictors of adolescent’s depression onset, especially for those requiring access to internal thoughts and feelings, parents provided a unique perspective accounting for additional information on the openness dimension. While adolescents’ self-reported personality measures are sufficient and reliable predictors of depression onsets, they might not capture the entire constructs of interest, as suggested by the current findings. Notably, parent-reported openness at the trait level and parent-reported depressivity at the facet level provided incremental predictive power above and beyond the corresponding self-reports in predicting depression onset. For future research and clinical utility, it may be worthwhile considering using informant-reports of these trait and facet as complementary measures on top of self-report to potentially improve clinical prediction of the onset of adolescent depression.

There are several limitations in the current study. First, we did not collect information on the facets of agreeableness and openness, as many prior studies did not find robust associations with depression (Kotov et al., 2010). However, it would be prudent to examine the effects of individual facets regardless of trait-level findings, given the higher degree of specificity demonstrated in facet analysis (Khoo & Simms, 2018). Second, depression onset was assessed using adolescents’ self-report, which potentially biased the study results in favor of self-reported personality measures as stronger predictors compared to parent-report. Third, we excluded adolescents with a history of diagnosable depression at baseline, which means that some individuals with strong personality vulnerabilities might not have been in the sample. Fourth, as adolescents spend increasingly more time in non-home contexts, parents may be less aware of youth’s behavior and social experiences than they were at earlier ages, reducing the accuracy of parent reports (Deros et al., 2018; Rausch et al., 2017). Fifth, baseline personality measures were taken as representative of enduring personal characteristics. While personality continuity is generally maintained from adolescence to early adulthood, it undergoes substantial changes in middle and old age (Roberts & DelVecchio, 2000; Roberts et al., 2001, 2006). The generalizability of how well personality predicts depression onset over years for different age groups needs to be further investigated in future research. Lastly, the present study focuses on females to avoid confounding due to sex differences (Kendler et al., 2002, 2006). Future research should examine whether these findings could be generalized to males. Similarly, the study sample consisted of primarily White adolescents residing in a specific location. It remains to be determined whether the current findings can be generalized to more racially, ethnically, and socioeconomically diverse groups.

The aim of the study was to examine the incremental predictive value of parent-reported personality as a predictor of first life-time onset depression in adolescent girls. We found that many of the personality predictors identified based on self-reported data were also significant predictors based on parent-report (e.g. neuroticism, depressivity), showing a high level of cross-informant consistency. In addition, parent-reported openness trait and depressivity facet exhibited unique predictive value. This contributes to our understanding of multi-informant approaches in developing more specific and tailored depression assessment and prevention strategies, whereby parent-reported personality measures potentially further inform us of adolescents at risks of developing first onsets depression.

### Supplementary Information

Below is the link to the electronic supplementary material.

Supplementary file1 (DOCX 16 KB)

## Data Availability

The data could be made available upon request to author Professor Roman Kotov.
