# Supplementary material for: The Prospective Predictive Power of Parent-Reported Personality Traits and Facets in First-Onset Depression in Adolescent Girls
Source: Res Child Adolesc Psychopathol. 2024 Mar 19;52(8):1221–31. doi: 10.1007/s10802-024-01186-w (PMC11289305; doi:10.1007/s10802-024-01186-w)
Supplement: Supplementary file 1 — Supplementary file1 (DOCX 16 KB) [file 10802_2024_1186_MOESM1_ESM.docx]

**Supplementary Table 1**

*Baseline Personality Measures Comparisons Between Completers (n=442) and Attritors (n=108)*

| Personality Measures | | Self-report | | Parent-report | |
| --- | --- | --- | --- | --- | --- |
|  |  | (M*_completers_* - M*_attritors_*) | Cohen’s *d* | (M*_completers_* - M*_attritors_*) | Cohen’s *d* |
| Neuroticism | | -0.2* | -0.27 | -0.3** | -0.28 |
|  | Anxiousness | -0.2 | -0.19 | -0.2 | -0.20 |
|  | Depressivity | -0.3** | -0.33 | -0.3*** | -0.38 |
| Conscientiousness | | 0.1 | 0.18 | 0.3** | 0.33 |
|  | Achievement | 0.1 | 0.08 | 0.2* | 0.27 |
|  | Deliberateness | 0.1 | 0.10 | 0.1 | 0.12 |
|  | Dutifulness | 0.1* | 0.23 | 0.2** | 0.32 |
|  | Orderliness | 0.2* | 0.24 | 0.3** | 0.31 |
|  | Self-discipline | 0.2 | 0.20 | 0.3** | 0.34 |
| Extraversion | | 0.0 | -0.03 | 0.1 | 0.13 |
|  | Ascendence | 0.1 | 0.08 | 0.0 | 0.01 |
|  | Positive Emotionality | 0.0 | 0.07 | 0.2* | 0.27 |
|  | Sociability | 0.0 | 0.05 | 0.1 | 0.11 |
|  | Venturesomeness | 0.0 | 0.06 | 0.0 | -0.04 |
| Agreeableness | | 0.1 | 0.19 | 0.2* | 0.28 |
| Openness | | 0.1 | 0.19 | 0.1 | 0.14 |

**p*<.05, ***p*<.01, ****p*<.001
